# Genome-wide association study of fish oil supplementation on lipid traits in 81,246 individuals reveals new gene-diet interaction loci

**DOI:** 10.1371/journal.pgen.1009431

**Published:** 2021-03-24

**Authors:** Michael Francis, Changwei Li, Yitang Sun, Jingqi Zhou, Xiang Li, J. Thomas Brenna, Kaixiong Ye

**Affiliations:** 1 Institute of Bioinformatics, University of Georgia, Athens, Georgia, United States of America; 2 Department of Epidemiology, Tulane University School of Public Health and Tropical Medicine, New Orleans, Louisiana, United States of America; 3 Department of Genetics, University of Georgia, Athens, Georgia, United States of America; 4 Division of Nutritional Sciences, Cornell University, Ithaca, New York, United States of America; 5 Department of Pediatrics, Dell Pediatric Research Institute, University of Texas at Austin, Austin, Texas, United States of America; 6 Departments of Nutrition and Chemistry, University of Texas at Austin, Austin, Texas, United States of America; Newcastle University, UNITED KINGDOM

## Abstract

Fish oil supplementation is widely used for reducing serum triglycerides (TAGs) but has mixed effects on other circulating cardiovascular biomarkers. Many genetic polymorphisms have been associated with blood lipids, including high- and low-density-lipoprotein cholesterol (HDL-C, LDL-C), total cholesterol, and TAGs. Here, the gene-diet interaction effects of fish oil supplementation on these lipids were analyzed in a discovery cohort of up to 73,962 UK Biobank participants, using a 1-degree-of-freedom (1df) test for interaction effects and a 2-degrees-of-freedom (2df) test to jointly analyze interaction and main effects. Associations with *P* < 1×10^−6^ in either test (26,157; 18,300 unique variants) were advanced to replication in up to 7,284 participants from the Atherosclerosis Risk in Communities (ARIC) Study. Replicated associations reaching 1df *P* < 0.05 (2,175; 1,763 unique variants) were used in meta-analyses. We found 13 replicated and 159 non-replicated (UK Biobank only) loci with significant 2df joint tests that were predominantly driven by main effects and have been previously reported. Four novel interaction loci were identified with 1df *P* < 5×10^−8^ in meta-analysis. The lead variant in the *GJB6*-*GJB2*-*GJA3* gene cluster, rs112803755 (A>G; minor allele frequency = 0.041), shows exclusively interaction effects. The minor allele is significantly associated with decreased TAGs in individuals with fish oil supplementation, but with increased TAGs in those without supplementation. This locus is significantly associated with higher *GJB2* expression of connexin 26 in adipose tissue; connexin activity is known to change upon exposure to omega-3 fatty acids. Significant interaction effects were also found in three other loci in the genes *SLC12A3* (HDL-C), *ABCA6* (LDL-C), and *MLXIPL* (LDL-C), but highly significant main effects are also present. Our study identifies novel gene-diet interaction effects for four genetic loci, whose effects on blood lipids are modified by fish oil supplementation. These findings highlight the need and possibility for personalized nutrition.

## Author summary

We utilized the unprecedentedly large genotype and phenotype dataset in the UK Biobank to perform a genome-wide association study (GWAS) which accounts for the interplay between genotype and dietary intake. We examined the interaction effects of fish oil supplementation on levels of blood lipids (LDL-C, HDL-C, TAGs, and total cholesterol). Our findings were replicated in the Atherosclerosis Risk in Communities (ARIC) Study. We found that at the genetic variant rs112803755 (A>G), the minor allele (G) is associated with a decrease in TAGs among individuals with fish oil supplementation, but is associated with an increase in TAGs among those without supplementation. In other words, only individuals carrying the minor allele benefit from fish oil supplementation in reducing TAG levels. We further analyzed rs112803755 with functional genomics data from the Genotype-Tissue Expression (GTEx) project to identify potential target genes, and found a connexin coding gene which has been previously reported to respond to cellular omega-3 levels. This research suggests that inter-personal variation in TAG response to fish oil supplementation is in part explained by genotype, and that fish oil dose adjustment based on genotype should be investigated as a means to protect against cardiovascular disease risk.

## Introduction

Dyslipidemia, characterized by imbalances in low-density lipoprotein cholesterol (LDL-C), high-density lipoprotein cholesterol (HDL-C), and triglycerides (TAGs), is a common predictive factor for metabolic conditions such as cardiovascular disease and type 2 diabetes [1,2]. Use of dietary supplements in lieu of xenobiotic pharmaceuticals for the management of dyslipidemia may produce comparable benefits with fewer side effects [1,2]. In particular, the omega-3 long-chain polyunsaturated fatty acids (n-3 LCPUFAs) eicosapentaenoic acid (EPA) and docosahexaenoic acid (DHA) supplied by fish oil supplements are an effective treatment for hypertriglyceridemia, though results are mixed for LDL-C and HDL-C [3–5]. Genetic polymorphisms have been consistently associated with intra- and inter-population differences in levels of LDL-C, HDL-C, total cholesterol, and TAGs [6,7]. Gene-environment interactions (GEIs; specifically, gene-diet interactions) between n-3 LCPUFAs and genetic variants have been reported, though few have been replicated, likely due to small sample sizes and inconsistencies in study designs such as study length and supplement dosage [8]. Studies of GEIs may reveal novel genetic loci that are otherwise obscure in conventional main-effect-only association studies, and may identify genetic loci whose phenotypic effects are modifiable by specific environmental exposures. Further identification of these GEIs may help explain both missing heritability in lipid biomarker traits [9], and heterogeneity of individual lipid response to fish oil supplementation [8,10–13].

To identify genomic factors which interact with n-3 LCPUFAs supplementation to affect levels of blood lipids, we performed a genome-wide association study (GWAS) among participants of the large UK Biobank cohort [14]. We used only participants whose genetic ethnic grouping is Caucasian, the largest sample available, to avoid population stratification [15]. The focus on single-ancestry groups is particularly important in studies related to LCPUFAs because their metabolic genes have been shown to undergo genetic adaptation to local diets in multiple geographical regions, and exhibit population-specific allele frequency patterns [16,17]. We used both the traditional 1-degree-of-freedom (1df) interaction test and a 2-degrees-of-freedom (2df) joint test to evaluate interactions between genetic variants and fish oil supplementation on blood lipid phenotypes. The 2df joint test evaluates single nucleotide polymorphism (SNP) main effects and interaction effects jointly, and therefore has higher power to detect SNPs with moderate main effects and moderate interaction effects that would otherwise be missed in the 1df test [18–20]. This method has recently been employed to examine the GEIs of these lipid traits with smoking [21], and sleep duration [22]. We further confirmed promising UK Biobank findings in a US cohort, the Atherosclerosis Risk in Communities Study (ARIC). Replicated SNPs were utilized in a meta-analysis of these studies to reveal new gene-diet interaction loci.

## Results

### Cohort demographics

Stage 1 discovery analyses were performed in up to 73,962 genetically Caucasian UK Biobank participants (S1 Table). Approximately 15.8% of these participants answered yes to taking fish oil supplements on dietary questionnaires at two time points taken between one and five years apart. The percentage male, mean age and BMI of UK Biobank participants were ~46.6%, 55.6 ± 7.9 (±1 SD) years old, and 27.0 ± 4.6 kg/m^2^, respectively. Stage 2 (replication) analyses were performed in up to 7,284 white participants in the ARIC cohort study. Approximately 1.4% of these participants answered yes to taking fish oil at one time point during their primary assessment. The percentage male, mean age and BMI of ARIC participants were ~47.0%, 54.3 ± 5.7 years old, and 26.9 ± 4.7 kg/m^2^, respectively.

### Gene-diet interaction GWAS

**Fig 1:**
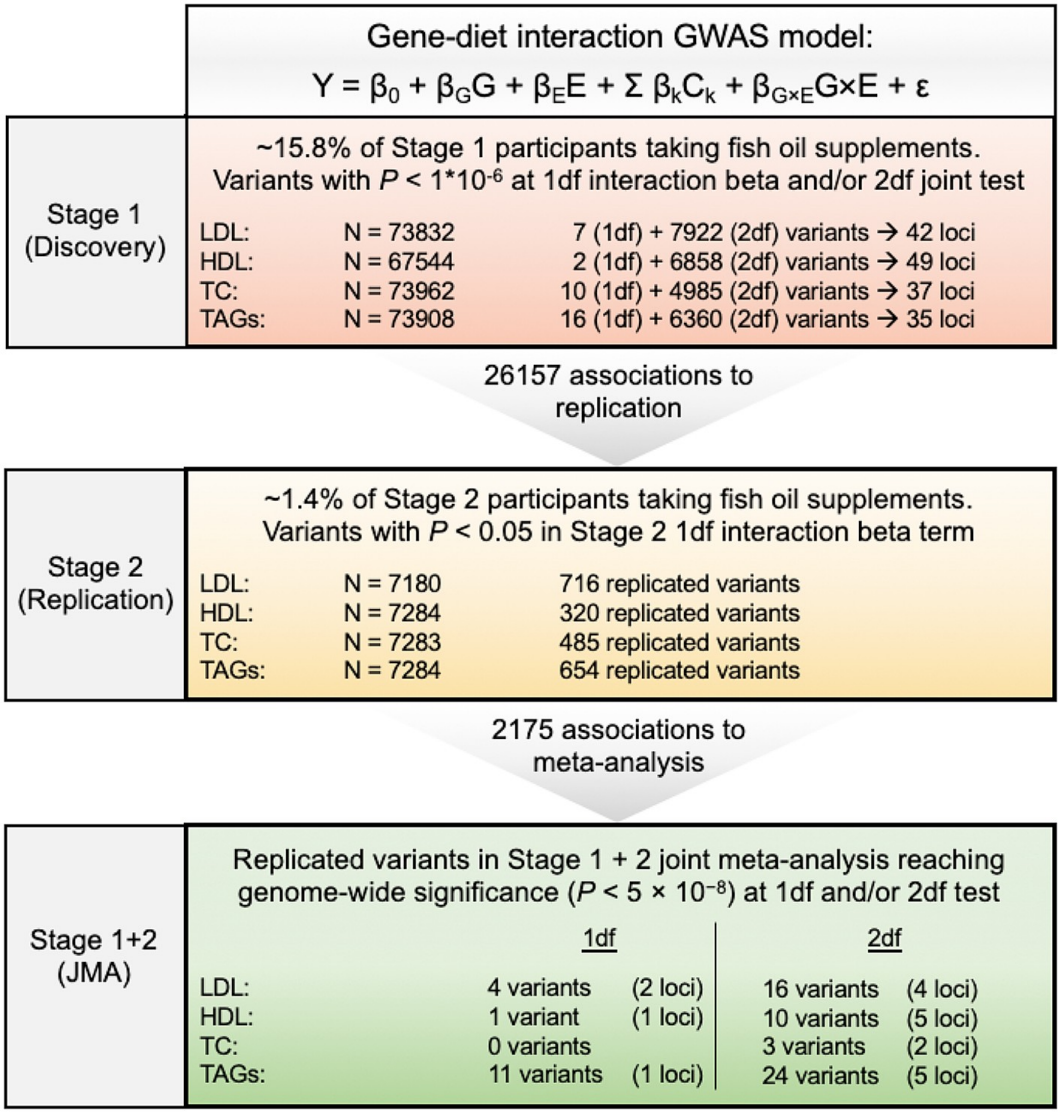
Overview of the analysis performed in this study. A three-stage discovery, replication, and meta-analysis process was used to identify significant variants. Stage 1 revealed 26,157 associations with 1df and/or 2df *P* < 1×10^−6^ in a cohort of up to 73,962 participants. Of these associations, 2,175 were replicated in a cohort of up to 7,284 participants. In meta-analysis, 4 1df loci (Table 1) and 16 2df loci (13 additional loci, Table 2) reached the genome-wide significance of *P* < 5 × 10^−8^. TC, total cholesterol; TAGs, triglycerides.

A three-stage discovery, replication, and meta-analysis approach for identification of significant GWAS loci was adopted for the blood lipid phenotypes LDL-C, HDL-C, total cholesterol, and TAGs (Fig 1). Genomic control (GC) correction was applied during Stage 1 2df *P*-value calculation and Stage 3 2df *P*-value calculation; lambda (λ) values after GC correction were 1. GC values of 1df *P*-values for Stage 1 and Stage 3 were < 1, therefore GC correction was not necessary.

Variants with 1df or 2df *P* < 1×10^−6^ in a gene-fish-oil interaction GWAS model (Eq (1)) were selected for replication (S2 Table). For the four lipid traits, LDL-C, HDL-C, total cholesterol, and TAGs, 26,157 associations (18,300 unique variants) met this criterion (S1 and S2 Figs).

Stage 2 replication analyses were performed in up to 7,284 white participants in the ARIC cohort (S1 Table). A gene-fish-oil interaction GWAS model was performed. Variants passed from Stage 1 with 1df *P* < 0.05 were considered as replicated. Of the 26,157 associations from Stage 1, a total of 2,175 associations (1763 unique variants) for the lipid traits were replicated (S3 Table) and passed to the meta-analysis step. There were also 17,259 associations (12,440 unique variants, 85 unique loci; S4 and S5 Tables) which reached genome-wide significance in Stage 1 (*P* < 5 × 10^−8^), but were not replicated in Stage 2, and therefore not sent to meta-analysis. All of the 85 lead variants had significant 2df joint test *P*-values, and none of their 1df interaction *P*-values approached significance, suggesting these variants influence lipid traits predominantly through main effects.

**Fig 2:**
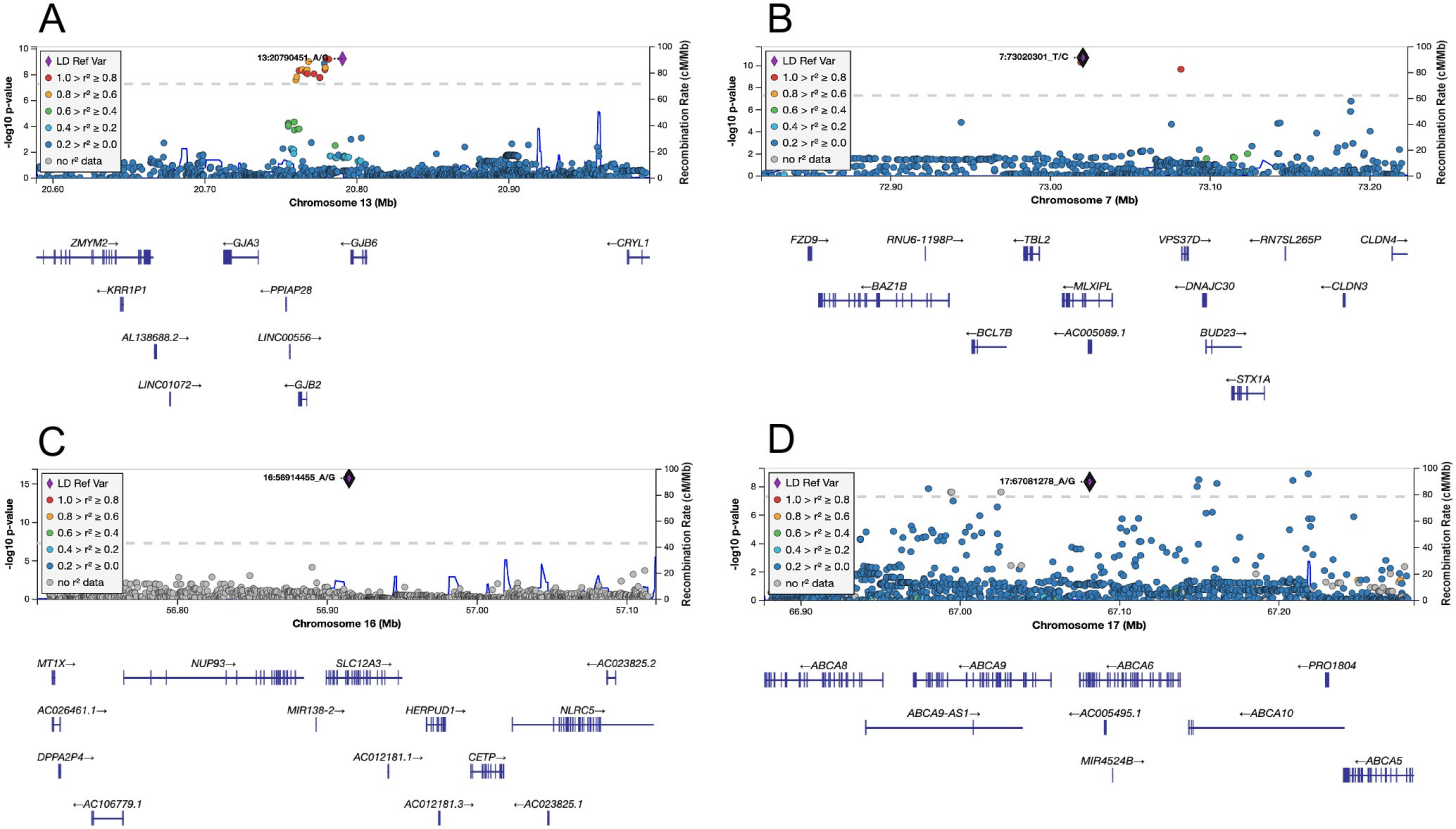
LocusZoom for genome-wide significant (*P* < 5 × 10^−8^) replicated gene-fish oil interaction loci. (A) rs112803755 × fish oil and TAGs, stage 1 + 2 1df tests (n = 81,192). (B) rs799157 × fish oil and LDL-C, stage 1 + 2 1df tests (n = 81,012). (C) rs148931404 × fish oil and HDL-C, stage 1 + 2 1df tests (n = 74,824). (D) rs77542162 × fish oil and LDL-C, stage 1 + 2 1df tests (n = 81,012).

Meta-analysis of Stage 1 and Stage 2 results for both 1df and 2df tests were performed for each blood lipid phenotype. Significant variants were defined in meta-analysis as those meeting the genome-wide significance threshold in their 1df or 2df tests (*P* < 5 × 10^−8^). This revealed 16 novel and significant 1df associations (4 unique loci; Table 1) and 53 significant 2df associations (11 unique loci; Table 2 and S6 Table). One variant, rs112803755 (*GJB6*; A>G; minor allele frequency (MAF) = 0.0410) had a significant 1df interaction term and no significant 2df or main effects terms. In the discovery cohort, the minor allele of SNP rs112803755 is associated with a strong decrease in TAGs among those taking fish oil supplements (β_G(E=1)_ = -0.12 mmol/L, *P* = 5.59×10^−5^), but is suggestively associated with a mild increase in TAGs for those without supplementation (β_G(E=0)_ = 0.030 mmol/L, *P* = 0.024), resulting in a significant interaction effect (1df *P* = 1.95×10^−7^). There is no association between the SNP main effects and TAGs in the UK Biobank (β_G_ = 0.0063 mmol/L, *P* = 0.60) if not considering the interaction effect. Meta-analysis revealed that the interaction effect at this SNP reaches genome-wide significance (1df *P* = 5.65 × 10^−10^). Three additional variants have both significant 1df interaction and 2df joint test *P*-values in the meta-analysis: rs799157 (*MLXIPL*; C>T; MAF = 0.0407) with LDL-C, rs77542162 (*ABCA6*; A>G; MAF = 0.0218) with LDL-C, and rs148931404 (*SLC12A3*; G>A; MAF = 0.0221) with HDL-C (Table 1 and Fig 2). In the discovery cohort, the minor allele of SNP rs799157 is associated with an increase in LDL-C (β_G_ = 0.057 mmol/L, *P* = 3.33×10^−8^) after adjusting for fish oil supplementation status and other covariates. Less significant associations were observed in the stratified groups with fish oil supplementation (β_G(E=1)_ = 0.087 mmol/L, *P* = 8.14×10^−4^) and in those without (β_G(E=0)_ = 0.052 mmol/L, *P* = 5.36×10^−6^). Meta-analysis confirmed the presence of main effect and revealed an interaction effect (1df *P* = 1.92×10^−11^, 2df *P* = 1.93×10^−33^). Similarly, SNP rs77542162 is associated with an increase in LDL-C in the overall discovery cohort (β_G_ = 1.41 mmol/L, *P* = 5.40×10^−23^), in those without (β_G(E=0)_ = 1.50, *P* = 4.24×10^−21^) and with (β_G(E=1)_ = 1.11, *p* = 1.55×10^−3^) fish oil supplementation. Meta-analysis revealed genome-wide significance in both tests (1df *P* = 4.48×10^−9^, 2df *P* = 6.58×10^−63^). For HDL-C, there is only one SNP, rs148931404, that reaches genome-wide significant 1df P-value (1.82×10^−16^) in the meta-analysis. It is associated with an increase in HDL-C in the overall discovery cohort (β_G_ = 0.049 mmol/L, *P* = 2.70×10^−16^), in those without (β_G(E=0)_ = 0.045 mmol/L, *P* = 5.67×10^−12^) and with (β_G(E=1)_ = 0.071 mmol/L, *P* = 3.49×10^−6^) fish oil supplementation. The three variants with both significant 1df and 2df *P*-values are mainly driven by main effects, as reflected by the much more significant 2df *P*-values and the consistent associations across subgroups in UK Biobank. All four loci have been previously found to be associated with the corresponding lipid. Overall, we unraveled novel gene-fish oil interaction effects for four previously known lipid-associated genetic loci.

**Table 1:** Loci with significant interaction between fish oil supplementation and blood lipid levels. Listed variants represent the lead association within a 1 Mb region for 1df tests of variant × fish oil interaction after meta-analysis. The name of the nearest gene is listed with each lead variant. Bold *P*-values indicate meeting the genome-wide significance threshold of *P* < 5 × 10^−8^. Main effect *P*-values are calculated using Stage 1 (UK Biobank) participants only, and without interaction (Eq (2); stratified for exposure groups as in Eq (3)). Effect, beta coefficient of the minor allele dose term (β_G_ in Eq (1)); MAF, minor allele frequency; SE, standard error; Int effect, beta coefficient of the interaction term (β_G×E_G×E in Eq (1)). Lipid traits were measured in mmol/L.

Stage 1 + 2 Meta-analysis genome-wide significant interaction loci													Stage 1			
hg19 chr:pos	rsID (nearest gene)	Minor (effect) allele (avg freq)	Reference allele	Stage1 MAF / Stage 2 MAF	Lipid trait	n	Effect	SE	Int Effect	Int SE	1df interaction *P*-value	2df joint *P*-value	n	Adj. Main effect *P*-value	Main effect *P*-value (E = 0)	Main effect *P*-value (E = 1)
13:20790451	rs112803755 (*GJB6*: ~5650 bp downstream)	g (0.0410)	a	0.0416 / 0.0352	TAGs	81192	0.04272	0.0447	-0.3084	0.1048	**5.65E-10**	0.01247	73908	0.6007	2.42E-02	5.59E-05
7:73020301	rs799157 (*MLXIPL*: Synonymous Variant)	t (0.0407)	c	0.0435 / 0.0112	LDL	81012	0.07162	0.05678	1.459	0.1371	**1.92E-11**	**1.93E-33**	73832	**3.33E-08**	5.36E-06	8.14E-04
17:67081278	rs77542162 (ABCA6: Missense Variant)	g (0.0218)	a	0.0226 / 0.0134	LDL	81012	0.1741	0.06495	-1.900	0.1224	**4.48E-09**	**6.58E-63**	73832	**5.40E-23**	**4.24E-21**	1.55E-03
16:56914455	rs148931404 (*SLC12A3*: Intron Variant)	a (0.0221)	g	0.0226 / 0.0172	HDL	74824	0.05594	0.02328	0.7734	0.04719	**1.82E-16**	**5.04E-91**	67544	**2.70E-16**	**5.67E-12**	3.49E-06

**Table 2:** Loci with significant 2df joint test between fish oil supplementation and blood lipid levels. Listed variants represent the lead association within a 1-Mb region for 2df tests of variant × fish oil interaction after meta-analysis. The name of the nearest gene is listed with each lead variant. Bold *P*-values indicate meeting the genome-wide significance threshold of *P* < 5 × 10^−8^. Main effect *P*-values are calculated using Stage 1 (UK Biobank) participants only, and without interaction (Eq (2); stratified for exposure groups as in Eq (3)). Effect, beta coefficient of the minor allele dose term (β_G_ in Eq (1)); MAF, minor allele frequency; SE, standard error; Int effect, beta coefficient of the interaction term (β_G×E_G×E in Eq (1)). Lipid traits were measured in mmol/L.

Stage 1 + 2 Meta-analysis replicated genome-wide significant loci													Stage 1			
hg19 chr:pos	rsID (nearest gene)	Minor (effect) allele (avg freq)	Reference allele	Stage1 MAF / Stage 2 MAF	Lipid trait	n	Effect	SE	Int Effect	Int SE	1df interaction *P*-value	2df joint *P*-value	n	Adj. Main effect *P*-value	Main effect *P*-value (E = 0)	Main effect *P*-value (E = 1)
6:34094919	rs115675705 (*GRM4*: Intron Variant)	g (0.0357)	a	0.0367 / 0.0268	HDL	74824	-0.03882	0.01795	-0.3348	0.04479	0.00873	**1.23E-19**	67544	**3.98E-09**	**3.43E-09**	2.24E-01
8:19722204	rs117860853 (*LPL*: ~217kb upstream)	a (0.0135)	g	0.0137 / 0.0116	HDL	74824	-0.08003	0.02691	-0.4139	0.05311	1.48E-02	**5.46E-28**	67544	**5.72E-24**	**3.47E-22**	3.00E-03
11:116916060	rs144018203 (*SIK3*: Intron Variant)	c (0.0102)	g	0.0107 / 0.0052	HDL	74824	-0.05472	0.03791	0.4387	0.05912	0.04122	**1.03E-16**	67544	**6.21E-17**	**1.16E-13**	1.00E-04
17:42061277	rs147438979 (*PYY*: Intron Variant)	c (0.011)	g	0.0116 / 0.006	HDL	74824	-0.06327	0.02952	-0.3336	0.05753	4.18E-02	**2.75E-16**	67544	**3.03E-09**	**1.78E-08**	5.58E-02
1:55583210	rs530804537 (USP24: Intron Variant)	a (0.011)	g	0.011 / 0.0012	LDL	81012	-0.07453	0.08869	-1.133	0.1360	0.1325	**2.68E-31**	73832	**2.01E-22**	**6.23E-21**	4.72E-03
19:45198060	rs112952132 (*LOC107985305*: Intron Variant)	t (0.01)	c	0.01 / 0.0101	LDL	81012	-0.2051	0.08381	-1.055	0.1329	2.77E-02	**7.58E-34**	73832	**9.49E-21**	**8.44E-18**	1.45E-01
7:72921771	rs117788606 (*BAZ1B*: Intron Variant)	c (0.0112)	t	0.0120 / 0.0032	TAGs	81192	-0.06165	0.09183	1.012	0.1464	2.67E-04	**3.93E-16**	73908	6.68E-07	7.64E-07	2.67E-01
8:19768150	rs142084074 (*LOC107986921*: Intron Variant)	a (0.015)	g	0.0150 / 0.0148	TAGs	81192	-0.1182	0.07241	0.911	0.1319	2.72E-02	**3.153E-12**	73908	**1.95E-15**	**1.42E-12**	2.84E-04
11:116916060	rs144018203 (*SIK3*: Intron Variant)	c (0.0103)	g	0.0108 / 0.0052	TAGs	81192	0.2531	0.1359	-0.9079	0.1735	0.008127	**2.48E-09**	73908	**3.50E-56**	**1.31E-50**	3.69E-07
15:43820717	rs55707100 (*MAP1A*: Missense Variant)	t (0.0249)	c	0.0247 / 0.0273	TAGs	81192	0.1201	0.05043	0.6116	0.1122	0.01044	**2.36E-13**	73908	**5.77E-21**	**2.03E-17**	5.18E-05
19:19365178	rs141844019 (*HAPLN4*: 500B Downstream Variant)	t (0.0104)	c	0.0109 / 0.0054	TAGs	81192	-0.06922	0.09157	1.637	0.1366	1.64E-06	**4.27E-53**	73908	3.91E-07	1.59E-06	1.06E-01
1:55583210	rs530804537 (*USP24*: Intron Variant)	a (0.0109)	g	0.0113 / 0.0073	Tot. Chol.	81245	-0.05886	0.1084	-1.057	0.1603	0.1002	**2.24E-20**	73962	**1.19E-17**	**9.04E-17**	1.88E-02
17:67081278	rs77542162 (*ABCA6*: Missense Variant)	g (0.0218)	a	0.0226 / 0.0135	Tot. Chol.	81245	0.1599	0.06831	-1.587	0.1261	4.58E-07	**1.80E-41**	73962	**3.06E-11**	**4.45E-11**	1.10E-01

There are 11 unique genetic loci whose 2df joint test *P*-values reached the genome-wide significance cutoff (*P* < 5 × 10^−8^) but their 1df interaction test *P*-values did not. For instance, a SNP upstream of *LPL* rs117860853 is associated with a decrease in HDL-C in the overall discovery cohort (β_G_ = -0.078 mmol/L, *P* = 5.72×10^−24^), in those without (β_G(E=0)_ = -0.081 mmol/L, *P* = 3.47×10^−22^) and with fish oil supplementation (β_G(E=1)_ = -0.063 mmol/L, *P* = 3.00×10^−3^). Meta-analysis revealed that this SNP has a significant main effect but no interaction effect (1df *P* = 0.015, 2df *P* = 5.46×10^−28^). Notably, two loci have 1df interaction test *P*-values that are close to the genome-wide significance level. SNP rs141844019, downstream of *HAPLN4*, has a suggestive interaction effect on TAGs (β_G×E_ = 1.64 mmol/L, *P* = 1.64×10^−6^), while SNP rs77542162, a missense variant of *ABCA6*, may have an interaction effect on total cholesterol (β_G×E_ = -1.59 mmol/L, *P* = 4.58×10^−7^). All these significant 2df replicated loci (Tables 1 and 2) were within 1 Mb of one or more previously reported loci associated with the same blood lipid phenotype and are therefore not reported as novel.

### rs112803755 modifies the effect of fish oil on TAGs

**Fig 3:**
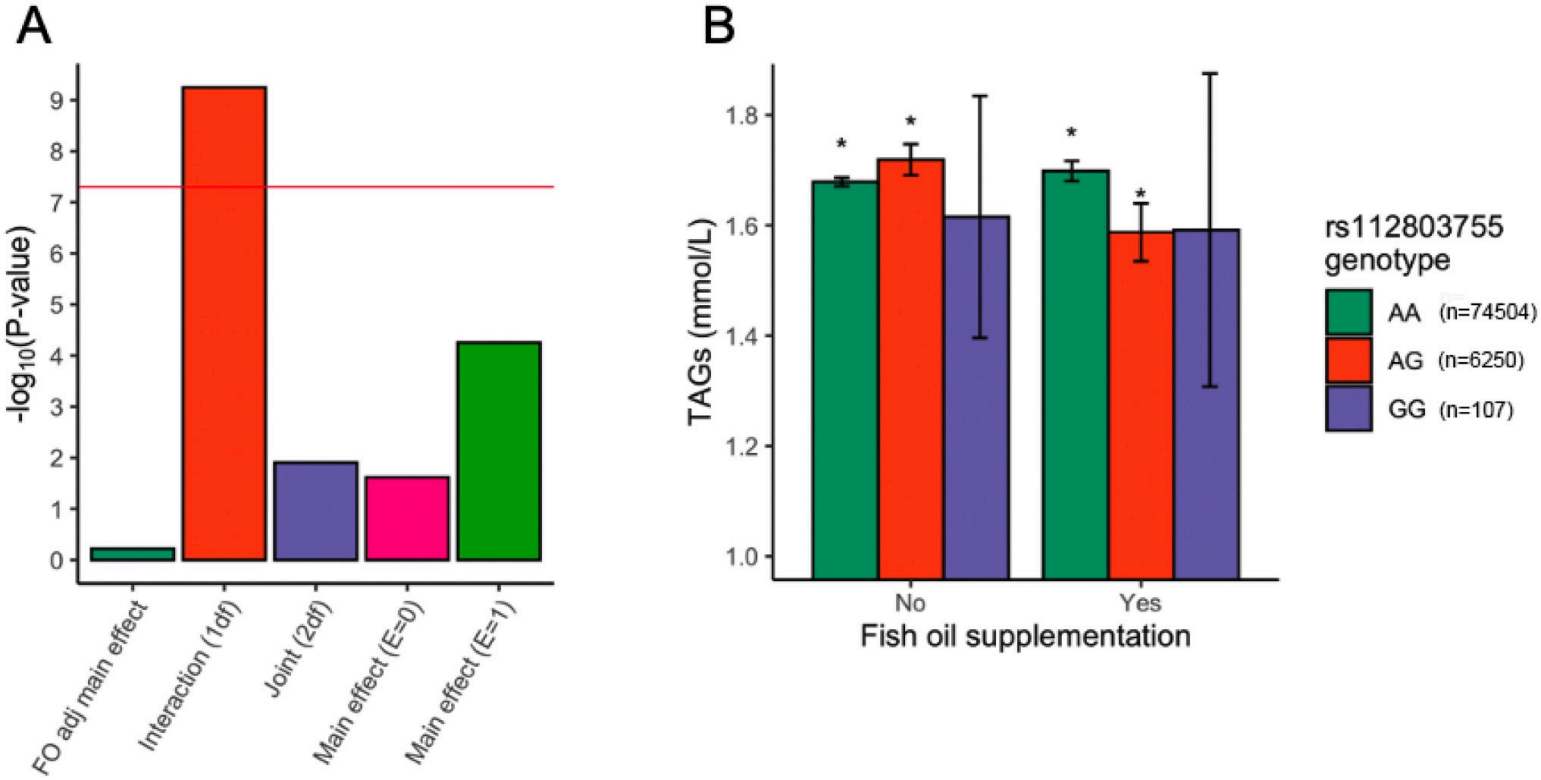
Significant results for the replicated interaction locus with lead SNP rs112803755. (A) rs112803755 *P*-values in five regression models. The red line is the negative log_10_-transformed genome-wide significance of 5 × 10^−8^. (B) Triglyceride lowering effect of fish oil supplementation on rs112803755 heterozygotes. Levels of TAGs stratified by genotypes at rs112803755 and fish oil supplementation status. Error bars show 95% confidence intervals. Exact numbers and sample sizes can be found in S7 Table.

Using TAG levels as a phenotype, the locus of 11 significant variants whose lead SNP is rs112803755 (*GJB6*: 5650 bp downstream; A>G; MAF = 0.0410) has a significant 1df interaction *P*-value (5.65 × 10^−10^), while its 2df joint P-value is not significant (*P* = 0.0124) (Fig 3A). Its fish-oil adjusted main effects model SNP term is not significant (*P* = 0.600), and in a stratified analysis the *P*-value is lower in the fish-oil supplementation exposure group (*P* = 5.59 × 10^−5^) than the non-supplementing group (*P* = 2.42 × 10^−2^) (Fig 3A). This evidence suggests that this locus is involved predominantly with interaction effects but not main effects.

The rs112803755 locus has significant TAG-lowering effect in those who supplement fish oil versus those who do not when considering AA vs. AG genotypes (Fig 3B and S7 Table). Since this variant has low MAF (~4.1%), homozygous individuals of GG genotype are rare. TAG levels were significantly higher in AG heterozygotes who did not take fish oil ($\Delta {\bar{\text{x}}}_{\text{TAGs}}=+0.04\;\text{mmol}/\text{L}$) versus those who did ($\Delta {\bar{\text{x}}}_{\text{TAGs}}=-0.111\;\text{mmol}/\text{L}$). However, with respect to rs112803755, while fish oil supplementation is associated with lower TAGs in heterozygous individuals, it has a slight opposite effect in AA homozygotes ($\Delta {\bar{\text{x}}}_{\text{TAGs}}=+0.0197\;\text{mmol}/\text{L}$; *P* = 0.0258).

### rs112803755 eQTL mapping

**Fig 4:**
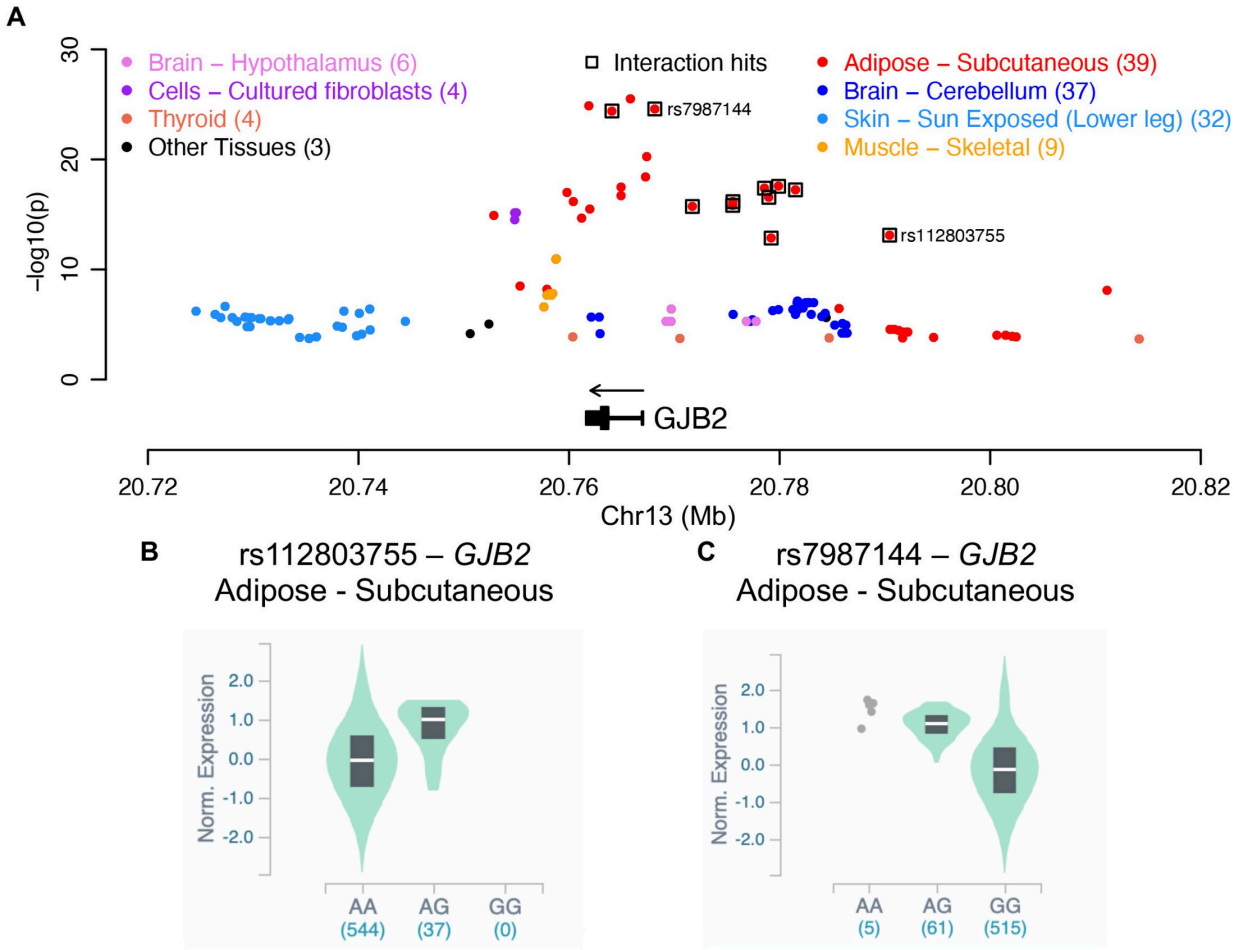
The gene-fish oil interaction locus with lead SNP rs112803755 overlaps eQTLs of *GJB2*. (A) Genetic variants significantly associated with the expression of *GJB2* as detected in the GTEx project. Colors indicate the tissues or cells. For variants with significant association in more than one tissues, the most significant p value is shown. The association of (B) rs112803755 and (C) rs7987144 with the expression of *GJB2* in subcutaneous adipose tissues.

To evaluate if regulation of gene expression is an underlying molecular mechanism for the interaction locus whose lead SNP is rs112803755 (Table 1), we interrogated the association of these genetic markers with expression levels of nearby genes using data from the Genotype-Tissue Expression (GTEx) project. For the 11 genetic markers in this locus with genome-wide significance of interaction with fish oil, all of them are exclusively associated with the expression of *GJB2*. Expression quantitative trait loci (eQTLs) for *GJB2* were found in multiple tissues but the strongest signals were observed in subcutaneous adipose, which overlap with the significant interaction signals (Fig 4A). rs112803755 is associated with *GJB2* expression in subcutaneous adipose (*P* = 7.7 × 10^−14^; Fig 4B), while another interaction SNP in this locus, rs7987144 (G>A; MAF = 0.0375), has an even stronger association (*P* = 2.6 × 10^−25^; Fig 4C). Both of these SNPs show increased *GJB2* expression with increased minor allele dosage. These eQTLs results indicate that regulatory variants of *GJB2* are likely responsible for the interaction signals at this locus.

## Discussion

In this gene-diet interaction GWAS, we identified and replicated novel interaction loci, in which fish oil supplementation affected levels of continuous lipid traits in a large Caucasian cohort. We found one locus, rs112803755, with a significant interaction effect but a non-significant main effect, suggesting that the presence of minor alleles at this locus can enhance the TAG-lowering effects of fish oil supplementation. We found three additional new significant interaction loci related to LDL-C and HDL-C levels, though these appear predominantly influenced by main effects (Table 1).

rs112803755 is found 5.65 kb downstream from *GJB6*, or alternatively 23.3 kb upstream from *GJB2*. It is also in high LD with variants found in the other genes in the *GJB6*-*GJB2*-*GJA3* gene cluster at 13q12.11 (Fig 2A). *GJB6*, *GJB2*, and *GJA3* are connexin (Cx) gap junction protein-coding genes that encode Cx30, Cx26, and Cx46, respectively. Cxs are responsible for forming hemichannels across gap junctions to enable the exchange of messenger molecules between adjacent cells. An n-6 LCPUFA, linoleic acid, has been shown to increase hemichannel activity of Cx26 in HeLa cells [23], and n-3 LCPUFAs lowered the expression of another connexin, Cx43, in rats with hypertriglyceridemia [24]. Genetic polymorphisms in another Cx gene are associated with protective effects on cardiovascular disease [25]. It is therefore plausible that changes in n-3 LCPUFA status induced by fish oil supplementation could interact with one Cx in this cluster to affect TAG levels. Although our analysis supports the likely presence of a regulatory variant, we also cannot rule out the existence of a causal coding variant.

rs799157 is a synonymous variant in exon 6 of *MLXIPL*, whose gene product is known as Carbohydrate-responsive element-binding protein (ChREBP). We found this variant has a significant interaction effect of fish oil on LDL-C. Variants in *MLXIPL* have previously been associated with changes in LDL-C and TAGs [26,27]. Intracellular levels of PUFAs are known to suppress ChREBP transactivity, though the molecular basis for this is not defined [28,29]. GTEx reveals that SNPs in this locus are significantly associated with increased expression of *TYW1B*. Specifically, increased minor allele dosage at rs799157, which we found to be associated with lower LDL-C levels, is most significantly associated with higher *TYW1B* expression in subcutaneous adipose tissue. *TYW1B* is a tRNA-yW synthesizing protein coding pseudogene involved in wybutosine synthesis, whose characteristics are not well-studied. This evidence suggests biological support for the ChREBP coding variant, while the regulatory variant for *TYW1B* is unlikely to be the causal variant.

rs148931404 is an intron variant of *SLC12A3* which we found to be associated with lower HDL-C levels. This gene has previously been associated with HDL-C in a large multi-ethnic GWAS [6]. *SLC12A3* encodes the sodium-chloride symporter protein. We did not find any plausible underlying biological mechanism for this variant. rs77542162 is a missense variant in *ABCA6* that we found to be associated with LDL-C. This variant has been reported in several GWAS studies in relation to LDL-C levels [6,7] and also in a 2df test joint GEI test with alcohol consumption [30]. Therefore, it is likely that this SNP is driven by main effects as *ABCA6* is thought to be regulated with macrophage lipid homeostasis [31].

Fish oil supplementation for treatment of hypertriglyceridemia has long been recognized [32]. Recent studies suggest that EPA and DHA have differential effects on HDL-C subfractions, but their overall effects on cardiometabolic lipid risk markers remain unresolved [33], despite dozens of human trials. Nearly all studies to date ignored genetic variants and focused on random cross sections of the population. Our unbiased study identified a variant modulating TAG levels, the only one of the lipid biomarker traits examined that is known to be clearly related to fish oil intake. Further, we identified variants modulating HDL-C and LDL-C, though these effects require further study. Overall, our study found no strong variants that may modulate LDL-C or HDL-C differentially between individuals based on fish oil supplementation status, thus supporting the hypothesis that EPA and DHA effects on these biomarkers are well represented by clinical trials that do not consider interaction with genotype. Our findings emphasize that a one-size-fit-all recommendation of fish oil supplementation to reduce TAG may not be appropriate. While individuals who are heterozygous (AG) at SNP rs112803755 experience a reduction in blood TAG when taking fish oil supplements, homozygotes of AA actually experience an increase. Based on the strong relationship between TAG and cardiovascular diseases, it is natural to hypothesize that the same genetic locus at *GJB2* might interact with n-3 LCPUFAs intakes to have differing effects on the risk of cardiovascular diseases. This is a promising hypothesis calling for direct tests in future studies.

Our study has several strengths and weaknesses. One strength granted by the UK Biobank is a large sample size with two data points taken several years apart for fish oil supplementation. This makes our discovery dataset quite robust and reduces the measurement error of our environmental exposure, which is an important consideration for GEI studies [34]. The ARIC data is less reliable, with only one fish oil data point. A weakness that we recognize is that other dietary quantities of n-3 and n-6 PUFAs are difficult to ascertain, and may interfere with the effects of fish oil. Another limitation of this study is that the ratio of samples in the discovery and replication cohorts is about 10:1. Currently, datasets which provide participant genotype data, fish oil supplementation use, and blood lipid measurements, are rare. Despite the difference in sample size between the UK Biobank and ARIC datasets, each is sufficiently powered to identify significant variants, with the exception of those which are rare or have low effect sizes. Previous gene-diet interaction studies of fish oil have had participants in the hundreds [8], and this is the largest fish oil interaction GWAS to date. One additional weakness is that there may be heterogeneity in the dosage of n-3 LCPUFAs provided by fish oil supplements. These limitations of exact nutrient quantification are present in most nutritional studies which rely on food frequency questionnaires and/or 24-hour recall surveys. Lastly, as in any other association study, ours is associative in nature and could not pinpoint the causal environmental exposure or the genetic variant [35]. We only examine one environmental exposure in this study, fish oil supplementation, which is correlated with many other lifestyle factors [36]. It is possible that other unexamined but correlated environmental factors drive the observed interaction effects, highlighting the need to perform interaction analysis with more environmental factors. However, our novel results make biological sense and many can be placed in a plausible mechanistic context. Finding significant interactions associated with the genes *GJB2* and *MLXIPL*, which have been shown to be regulated by PUFAs, is a validation of our approach.

Our study unravels novel gene-diet interaction effects for four genetic loci, whose effects on blood lipids are modified by fish oil supplementation. Such results lend further support to the practice of precision nutrition to catalyze nutrition science into meaningful and clinically relevant dietary suggestions [37]. Personalizing and optimizing fish oil supplementation recommendations based on a person’s unique genetic composition can improve our understanding of nutrition, and lead to significant improvements in human health and well-being. Once validated, these variants in *GJB2*, *SLC12A3*, *ABCA6*, and *MLXIPL*, will contribute to our understanding of how accounting for genetic differences can allow every person to implement their optimal nutrient intake. Accounting for interaction effects can also help us better understand biological processes leading to disease, and improve the accuracy of future risk prediction models.

## Methods

### Ethics statement

Use of participant data was approved by the University of Georgia Institutional Review Board, UK Biobank (Project ID 48818), and the National Center for Biotechnology Information. Participants of UK Biobank and the Atherosclerosis Risk in Communities Study (ARIC) have signed written consent forms authorizing the use of their medical and genetic data for use in research studies. All methods were performed securely and in accordance with ethical guidelines and regulations.

### Participants

UK Biobank is a prospective cohort study which recruited > 500,000 volunteer participants between 2006 and 2010 in England, Scotland and Wales. Biochemical, clinical, and genotype data were collected. ARIC is a prospective cohort study conducted in four U.S. communities, which began in 1987 and continued to 2007. ARIC participants were randomly selected from pre-defined populations to have medical, social, and demographic data collected. All participants were 40 to 70 years of age at the time of assessment. Participant characteristics can be found in S1 Table.

Participants were quality controlled on the following criteria: genetic ethnicity is Caucasian, used in PCA analysis, not an outlier for heterogeneity and missing genotype rate, no sex chromosome aneuploidy, does not have high degree of genetic kinship (ten or more third-degree relatives identified), and self-reported sex matches genetic sex. Additionally, we removed the minimum number of participants to eliminate all related pairs.

### Phenotypes

All continuous blood lipid measures are reported and analyzed in mmol/L. For stage 1 participants, lipid measures were collected during the UK Biobank Assessment Centres initial assessment from 2006–2010. Blood lipids were analyzed by direct aliquot assays in UK Biobank participants using a Beckman Coilter AU5800. LDL-C was measured by enzymatic protective selection analysis; HDL-C was measured by enzyme immunoinhibition analysis; total cholesterol was measured by CHO-POD analysis; TAGs were measured by GPO-POD analysis.

For ARIC participants, plasma was ultracentrifuged to obtain VLDL-free infranate. LDL-C was precipitated by addition of dextran sulfate and Mg^2+^ to separate an HDL-C supernate. HDL-C was re-precipitated with dextran sulfate and Mg^2+^, and separated by centrifugation. LDL-C levels were calculated using the Friedewald equation. TAGs and total cholesterol were processed and their levels measured by spectrophotometry as described in the ARIC manual for Lipid and Lipoprotein Determinations [38].

LDL-C was adjusted for those who self-reported the use of statins or lipid-lowering drugs as described in [19]; this adjustment was performed in 9,951 UK Biobank participants and 316 ARIC participants. No adjustments were made for other lipids.

### Fish oil supplementation status

Blood LCPUFA levels were not taken in UK Biobank or ARIC cohort studies. Because omega-3 content in dietary intake can vary significantly depending on animal feed quality (e.g. egg laying hens fed an omega-3 rich diet), as well as source (e.g. wild or farmed raised salmon) [39–42], and since neither dietary questionnaire specifies these details, we use fish oil consumption as a minimally confounded contributor to EPA and DHA consumption [43].

Dietary intake data for UK Biobank participants was taken at two time points approximately 3–4 years apart. Participants were asked of their supplement use, including fish oil, in their health and medical history questionnaire at the initial assessment, "Do you regularly take any of the following? (You can select more than one answer)" (f.6179). An online follow-up assessment which included the Oxford WebQ, a digital 24-hour dietary recall questionnaire, was completed by UK Biobank participants on a voluntary basis between 2011–2012 [44,45]. Participants self-reported their use of dietary supplements from the preceding 24 hours (f.20084). Those who answered yes to fish oil supplementation at both time points were coded as 1, those who answered no at both points were coded as 0, and those with different answers were excluded from our analysis (S3 Fig).

ARIC participants indicated their fish oil supplementation status at one time point during their primary assessment. Participants were asked “Do you regularly take fish oil? (Including omega-3 fatty acids, EPA, cod liver oil).” in the “Vitamin Survey Form” at the date of their primary assessment between 1985–2007.

### Covariates

Covariates used in our association analyses were age, sex, body mass index (BMI), weekly servings of oily fish, socioeconomic status measured by Townsend deprivation index, and the first ten genetic principal components. BMI is measured in kg/m^2^, and was transformed using ordered quantile normalization for ARIC participants. Weekly servings of oily fish were converted to ordinal variables ranging from 0 (none) to 5 (more than one serving per day). Genetic principal components were provided in the original genotype data of both cohorts.

### Genotype data

The first 50,000 UK Biobank participants of the full study cohort were genotyped using the Affymetrix UK BiLEVE Axiom array, and the remaining 450,000 participants were genotyped using the Affymetrix UK Biobank Axiom array; the two arrays are more than 95% similar in their variant content. Imputation and initial quality control of UK Biobank SNPs were performed by a collaborative group headed by the Wellcome Trust Centre for Human Genetics. We excluded autosomal SNPs with imputation quality score < 0.5, minor allele frequency (MAF) < 1%, missing genotype per individual > 5%, missing genotype per variant > 2%, or Hardy-Weinberg equilibrium (HWE) *P* < 1×10^−6^. After quality control, a total of 7,954,107 autosomal variants among 73,962 participants were included in the analyses. Our quality control and genotype file format conversions were performed using PLINK2 alpha-v2.3 [46–48].

ARIC participants were genotyped using the Affymetrix GeneChip SNP Array 6.0. Before imputation, quality control removed variants with missing rate > 10%, or MAF < 1%, and individuals with missing genotype rate > 80%. After quality control, genotypes were imputed to the ALL ancestry panel of the 1000 Genome Phase III integrate Release Version 5 [49] using MiniMac software [50]. After imputation, SNPs with *r*^2^ < 0.50, MAF < 1%, or HWE *P* < 1×10^−6^ were removed.

### Stage 1 analysis

Stage 1 analysis included up to 73,962 UK Biobank participants and up to 7,954,107 variants after quality control (S1 Table).

Interaction regression was performed for each variant using QuickTest (v1.2) according to the following fixed effects GWAS interaction model:

$$\text{Y}=\beta_{0}+\beta_{\text{G}}\text{G}+\beta_{\text{E}}\text{E}+\Sigma {\;\beta }_{\text{Ck}}{\text{C}}_{\text{k}}+\beta_{\text{G}\times \text{E}}\text{G}\times \text{E}+\varepsilon$$ (1)

where Y is a measure of lipid traits (LDL-C, HDL-C, total cholesterol, and TAGs), G is the effect variant count (0/1/2), E is a binary variable representing fish oil supplementation status (0/1), C_k_ are covariates, and G×E is the GEI term (S4 Fig). Regression coefficients and *P*-values were calculated using QuickTest normal mean method for expected genotype dosages; this method is implemented to reduce false positives [51]. Robust Huber sandwich estimates of the variance-covariance matrix were generated.

Main effects adjusted by E were calculated according to the fixed effects model:

$$\text{Y}=\beta_{0}+\beta_{\text{G}}\text{G}+\beta_{\text{E}}\text{E}+\Sigma {\;\beta }_{\text{Ck}}{\text{C}}_{\text{k}}+\varepsilon$$ (2)

Main additive variant effects, and variant effects stratified by (E) were also calculated using the generalized fixed effects model:

$$\text{Y}=\beta_{0}+\beta_{\text{G}}\text{G}+\Sigma {\;\beta }_{\text{Ck}}{\text{C}}_{\text{k}}+\varepsilon$$ (3)

These main effects models were performed using the same QuickTest normal mean method.

Joint *P*-values of main and interaction effects (β_G_ and β_G×E_) were calculated according to a 2df *χ*^2^ distribution which corrected for the determinant of the covariance matrix between these two terms [18]. Genomic control was applied to Stage 1 2df joint *P*-values for each lipid phenotype. Variants reaching *P* < 1 × 10^−6^ in either the 1df interaction test or 2df joint test were advanced to replication in Stage 2.

### Stage 2 analysis

Stage 2 analysis included up to 7,284 ARIC participants, and 48,608,505 variants (S1 Table). Participants were filtered on the basis of their ethnicity (white) only. Additional quality control on samples and genomic data (as in Stage 1) was not conducted, because these filters are meant to reduce the rate of false positives, which was not relevant for Stage 2 replication. Regression coefficients and *P*-values were calculated using QuickTest normal mean method. Variants advanced from Stage 1 which also had a *P* < 0.05 in the 1df interaction term in the ARIC cohort were advanced to joint meta-analysis between the two cohorts in Stage 1+2.

### Meta-analysis of stage 1+2

METAL meta-analysis software (2010-02-08) [52] was used to perform a meta-analysis of those associations with *P* < 1 × 10^−6^ in 1df interaction and/or 2df joint tests in Stage 1, and *P* < 0.05 in 1df interaction test in Stage 2 (patch provided by A. Manning to enable 2df GEI testing [18]; genome.sph.umich.edu/wiki/Meta_Analysis_of_SNPxEnvironment_Interaction). Stage 1+2 meta-analyses were performed using a weighted z-statistic by sample size [52]. Genomic control was applied to all meta-analyses as implemented by METAL. Associations exceeding the genome-wide significance threshold of *P* < 5 × 10^−8^ were passed to FUMA to identify the lead SNP for each locus.

### Identifying lead SNPs

Variants exceeding the genome-wide significance threshold of *P* < 5 × 10^−8^ were inputted to FUMA to identify independent loci and their lead SNPs [53]. Lead SNPs are defined as the SNP within a locus having the lowest *P*-value. UK Biobank release 2b 10k White British was used as the reference panel population. The maximum *P*-value cutoff was set to 0.05, and a first threshold of *r*^2^ ≥ 0.6 and second threshold of *r*^2^ ≥ 0.1 were used to define independent significant SNPs. The maximum distance between LD blocks to merge into a locus was < 1Mb.

### Identifying novel variants

For replicated and non-replicated variants with joint meta-analysis *P* < 5 × 10^−8^, GWAS Catalog [54] was used to identify novel variants. Gene-fish-oil interaction variants were checked in a literature search for their novelty. Variants within 1Mb from previously published variants associated with the same trait were considered to be non-novel.

### Additional analyses

The R package qqman v 0.1.4 was used to generate Manhattan plots and QQ plots [55]. Regional loci plots were made using LocusZoom [56]. Data analysis was conducted in R v3.6.1 [57]. The Genotype-Tissue Expression Project (GTEx) data used were obtained from the GTEx Portal on 04/29/20 [58].

## Supporting information

S1 Fig Manhattan plots for Stage 1 1df interaction term *P*-values and 2df joint test *P*-values for lipid traits. Plots show post-genomic control values.
(TIF)

S2 Fig QQ plots for Stage 1 1df interaction term *P*-values and 2df joint test *P*-values for lipid traits. Plots show post-genomic control values.
(TIF)

S3 Fig Fish oil supplementation taken at two time points. The number of UK Biobank participants who responded yes/yes, no/no, yes/no, and no/yes to the two dietary assessment time points at the initial assessment and in the 24-hour follow-up questionnaire are shown. Numbers reflect the total number of participants who answered in both assessments, but not the number of participants used in this study after quality control.
(TIF)

S4 Fig Visualization of the G×E interaction regression model. Y = β_0_ + β_G_G + β_E_E + Σ β_k_C_k_ + β_G×E_G×E + ε, where Y = phenotype, G = minor variant dosage (0/1/2 coding), E = environmental exposure, C_k_ = covariates, and G×E = interaction term. In this study, Y is a continuous lipid trait, and E is a binary variable representing the presence or absence of self-reported dietary fish oil supplementation.
(TIF)

S1 Table Participant characteristics. Participant characteristics, by blood lipid phenotype, for those included in GEI analyses for Stage 1 (UK Biobank) and Stage 2 (ARIC). Mean and standard deviation values are shown for blood lipid phenotypes and for applicable covariates.
(XLSX)

S2 Table Numbers of stage 1 significant variants. Variants which passed a significance threshold of *P* < 1e-06 in Stage 1 (UK Biobank) are counted here. Significance was assessed for both 1df interaction terms and 2df joint terms. Variant count and number of independent loci are shown, as well as unique variants with 1df and 2df tests.
(XLSX)

S3 Table Numbers of replicated variants. Variants which reached Stage 1 *P* < 1e-06 (in either 1df or 2df) and were found to have 1df *P* < 0.05 in Stage 2 interaction models.
(XLSX)

S4 Table Numbers of genome-wide significance loci in only Stage 1. Counts of variants and loci which met the significance threshold of P< 5e-08 in Stage 1 (in either 1df or 2df) but which were not replicated in Stage 2. Note that no Stage 1 1df *P*-values reached this threshold so all variants in this table refer to their 2df joint test *P*-values.
(XLSX)

S5 Table Non-replicated genome-wide significant Stage 1 variants. Full details for the loci counted in Table S4. Effect, beta coefficient of the minor allele dose term (β_G_ in Eq (1)); MAF, minor allele frequency; SE, standard error; Int effect, beta coefficient of the interaction term (β_G×E_G×E in Eq (1)). Lipid traits were measured in mmol/L. All *P*-values are calculated using Stage 1 (UK Biobank) participants only.
(XLSX)

S6 Table Numbers of genome-wide significance loci after meta-analyses. Counts of replicated results reaching genome-wide significance (*P* < 5e0−8) in Stage 1+2 meta-analyses. Significant variants determined by 1df *P*-values (top) and 2df *P*-values (bottom).
(XLSX)

S7 Table Data used in Fig 3B. Fish oil status, number of G alleles at rs112803755, mean triglycerides, sample size, standard deviation of triglycerides, and 95% confidence interval for combined participants from Stage 1 and Stage 2.
(XLSX)
