# Correction: Genome-wide association study of fish oil supplementation on lipid traits in 81,246 individuals reveals new gene-diet interaction loci

**DOI:** 10.1371/journal.pgen.1010735

**Published:** 2023-04-18

**Authors:** Michael Francis, Changwei Li, Yitang Sun, Jingqi Zhou, Xiang Li, J. Thomas Brenna, Kaixiong Ye

The Data Availability statement is incomplete. The complete statement should read:

Individual-level genetic and phenotypic data cannot be shared publicly because of participant privacy. Data are available from the UK Biobank Institutional Data Access / Ethics Committee (https://www.ukbiobank.ac.uk/register-apply/) with applications. The ARIC datasets used for the analyses in this manuscript were obtained from dbGaP through dbGaP accession study number phs000280.v3.p1. All summary statistics for Gene-Fish Oil Interactions are publicly available on figshare (https://doi.org/10.6084/m9.figshare.14171069.v1). Key computational scripts are available here: https://github.com/michaelofrancis/FishOil-Lipid-Interaction. All other relevant data are within the manuscript and its Supporting information files.

The Acknowledgments section is incomplete. The complete Acknowledgments should read:

The authors would like to thank all UK Biobank participants and administrators for data access. We also thank all Ye lab members for helpful discussions.

The Atherosclerosis Risk in Communities (ARIC) study has been funded in whole or in part with Federal funds from the National Heart, Lung, and Blood Institute, National Institute of Health, Department of Health and Human Services, under contract numbers (HHSN268201700001I, HHSN268201700002I, HHSN268201700003I, HHSN268201700004I, and HHSN268201700005I). The authors thank the staff and participants of the ARIC study for their important contributions. Funding for the ARIC Gene Environment Association Studies (GENEVA) was provided by National Human Genome Research Institute grant U01HG004402 (E. Boerwinkle). The datasets used for the analyses in this manuscript were obtained from dbGaP through dbGaP accession study number phs000280.v3.p1.
