# Supplementary figures and images for: Genome-wide association study of fish oil supplementation on lipid traits in 81,246 individuals reveals new gene-diet interaction loci
Source: PLoS Genet. 2021 Mar 24;17(3):e1009431. doi: 10.1371/journal.pgen.1009431 (PMC8021161; doi:10.1371/journal.pgen.1009431)

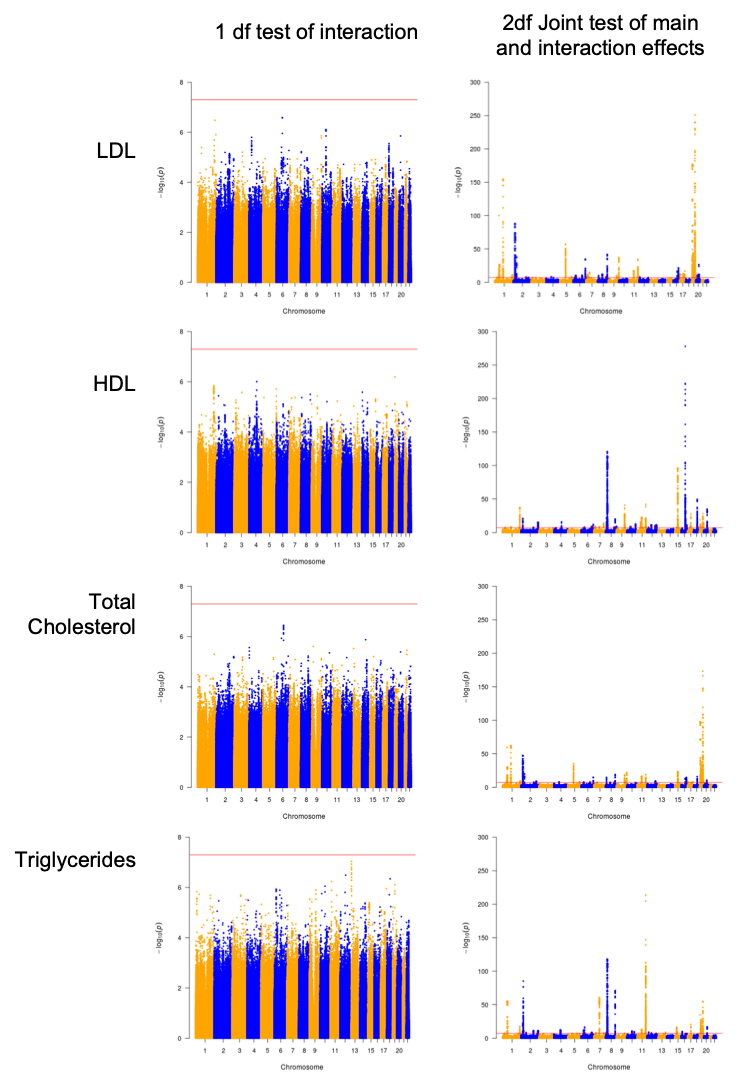

Supplement: S1 Fig — Plots show post-genomic control values. (TIF) [file pgen.1009431.s001.tif]

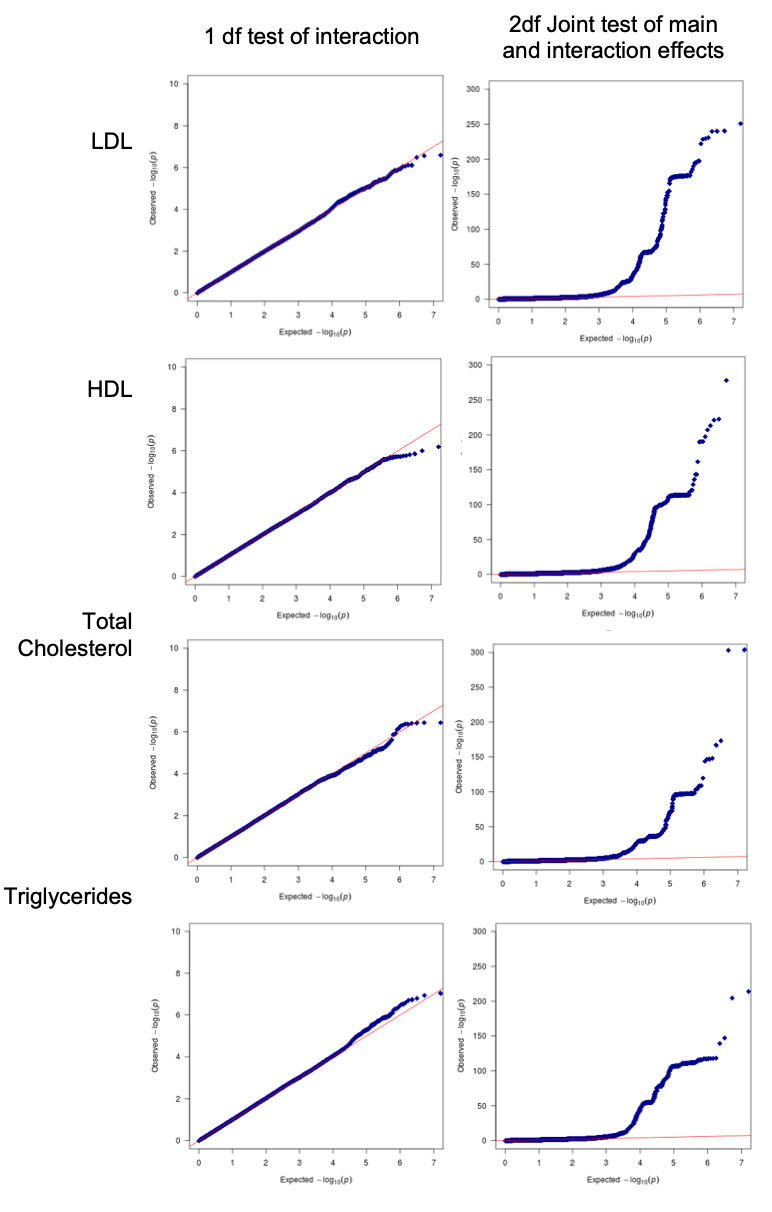

Supplement: S2 Fig — Plots show post-genomic control values. (TIF) [file pgen.1009431.s002.tif]

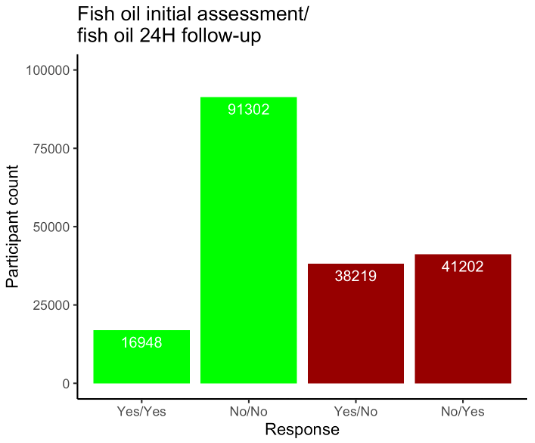

Supplement: S3 Fig — The number of UK Biobank participants who responded yes/yes, no/no, yes/no, and no/yes to the two dietary assessment time points at the initial assessment and in the 24-hour follow-up questionnaire are shown. Numbers reflect the total number of participants who answered in both assessments, but not the number of participants used in this study after quality control. (TIF) [file pgen.1009431.s003.tif]

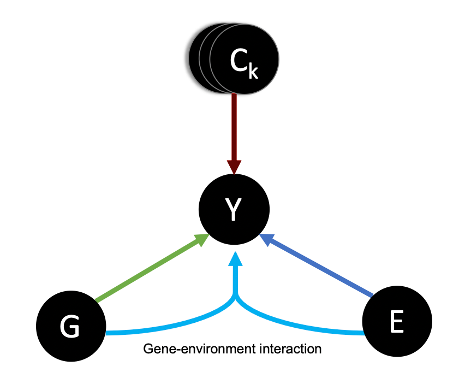

Supplement: S4 Fig — Y = β0 + βGG + βEE + Σ βkCk + βG×EG×E + ε, where Y = phenotype, G = minor variant dosage (0/1/2 coding), E = environmental exposure, Ck = covariates, and G×E = interaction term. In this study, Y is a continuous lipid trait, and E is a binary variable representing the presence or absence of self-reported dietary fish oil supplementation. (TIF) [file pgen.1009431.s004.tif]
